# Filoviruses: A Compendium of 40 Years of Epidemiological, Clinical, and Laboratory Studies

**DOI:** 10.3201/eid1512.091044

**Published:** 2009-12

**Authors:** Pierre Rollin

**Affiliations:** Centers for Disease Control and Prevention, Atlanta, Georgia, USA

**Keywords:** Filovirus, Marburg, Ebola, viruses, zoonoses, book review

In Filoviruses: A Compendium of 40 Years of Epidemiological, Clinical, and Laboratory Studies, Jens Kuhn presents a complete review of every paper published on the subject, as well as hundreds of unpublished reports. In addition, most of the world’s known experts on filoviruses contributed personal data and anecdotes. A CD-ROM with a searchable list of all quoted references (≈4,500) is a useful addition to the book.

The author details the history of all Marburg and Ebola outbreaks during the 40 years from the discovery of Marburg in 1967 to the latest Marburg outbreak in 2007 in Uganda, including difficult-to-find information. The clinical and pathologic presentations of Ebola and Marburg diseases in human and animal models contain a substantial number of black and white and color illustrations. With the range of the Ebola and Marburg viruses still unknown and the search for the animal reservoirs ongoing, antibody serosurveys in humans and animals have been conducted in most countries in Africa and in the Philippines after the Ebola Reston outbreaks; Kuhn dedicates a long chapter to this subject. All animal species tested are presented in tables but, more interestingly, also indexed at the end of the book. This information is particularly valuable for ecologists and epidemiologists searching for the reservoir of filoviruses.

Kuhn explains the structure and replication of the filoviruses, with the actual role of each gene of the virus from entry into the target cells to production of infectious virus. He lists diagnosis techniques and experimental treatments of the Marburg and Ebola diseases, from the traditional healers to the molecular antisenses RNA approaches. In the past 10 years, preexposure and postexposures vaccinations have resulted in tremendous progress in schedules, routes of administration, and more importantly, understanding mechanisms of action. Lack or efficacy of inactivated and live-attenuated constructs is reviewed of all testing in available animal models.

This book is not, and is not supposed to be, a critical review of the literature but is rather a compilation of all known information on filoviruses by subject matter experts that is presented for filovirologists. Nonspecialist virologists, scientists, epidemiologists, clinicians, and students interested in the subject will also find the book useful, but they will have to digest and analyze the information and then weigh the values and relevance of this incredible compendium of data.

